# It Is Not All about Being Sweet: Differences in Floral Traits and Insect Visitation among Hybrid Carrot Cultivars

**DOI:** 10.3390/insects11070402

**Published:** 2020-06-29

**Authors:** Ann Gaffney, Björn Bohman, Stephen R. Quarrell, Philip H. Brown, Geoff R. Allen

**Affiliations:** 1 Tasmanian Institute of Agriculture, University of Tasmania, Churchill Ave, Hobart 7005, Australia; ann.gaffney@alumni.sydney.edu.au (A.G.); stephen.quarrell@utas.edu.au (S.R.Q.); p.h.brown@cqu.edu.au (P.H.B.); geoff.allen@utas.edu.au (G.R.A.); 2 School of Molecular Sciences, The University of Western Australia, 35 Stirling Hwy, Perth 6009, Australia; 3 Department of Plant Protection Biology, Swedish University of Agricultural Sciences, Box 102, 23053 Alnarp, Sweden; 4 School of Health, Medical and Applied Sciences, Central Queensland University, Bundaberg 4670, Australia

**Keywords:** crop carrot, hybrid, honey bee, pollination

## Abstract

Cytoplasmically male-sterile (CMS) carrot cultivars suffer from low pollination rates. In this study, insect visitation varied more than eightfold between 17 CMS carrot cultivars in a field-based cultivar evaluation trial. The visitation rates of honey bees, nectar scarabs, muscoid flies, and wasps each significantly differed among these cultivars. No significant difference in visitation rates was observed among cultivars of different CMS type (brown-anther or petaloid) or flower colour, but cultivars of Berlicumer root type had significantly higher insect visitation rates than Nantes. Six cultivars were further compared in regard to selected umbel traits: as umbel diameter increased, so did the visitation of soldier beetles, while that of honey bees decreased. Finally, nectar of these six cultivars was analysed for sugar content, which revealed monosaccharides to be the most common sugars in all. There was high variation in the levels of sugars from individual umbellets but no significant difference in nectar sugar composition among cultivars, suggesting that nectar sugar composition is of minor importance regarding pollinator attraction to hybrid CMS carrot umbels.

## 1. Introduction

Carrot flowers are unattractive to honey bees (*Apis mellifera* Linnaeus), which often seek out other available forage [1]. Hybrid or cytoplasmically male-sterile (CMS) cultivars are particularly unattractive, as they do not provide pollen [2,3,4]. Such cultivars are also believed to be inferior in their morphology, scent, and nectar production to male fertile (MF) plants [5]. 

Several studies have reported species-selective pollinator visitation to hybrid carrot seed crops including pollinators such as *A. mellifera* [2,3,6,7,8], flies [9,10], *Megachile* bees [11], and other native bees [12], but there appear to be no reports on carrot pollinator assemblage level visitation to different CMS cultivars, despite strong indications of differences in pollinator attraction among cultivars [13,14,15]. 

Generally, there are many visual and olfactory floral traits, including morphology, colour, nectar composition, and floral volatiles, which separately or collectively make one flower easily distinguishable from another to a pollinator [16,17,18]. Furthermore, some pollinators such as honey bees are opportunistic foragers, whose tendencies for innate responses can be altered by learning, mainly via olfactory conditioning [19,20]. Manipulation of the chemistry in the colony has also been investigated, where the treatment of honey bee colonies with brood pheromone increased the foraging and yield of carrot seed in nearby fields [21].

The nectar quality and availability are of almost universal importance to all insect foragers, as it is one reward that encourages insects to return to a flower [22,23]. Most honey bee-pollinated plants have high levels of sucrose in their nectar [24,25]. Percival analysed the nectar content of 889 species of angiosperm and concluded that plants pollinated by honey bees tend to have high disaccharide/monosaccharide (fructose and glucose) ratios [26]. Nonetheless, crops well-visited by bees showed a relatively balanced distribution of sugars, and it was concluded that bees show no direct preference for sucrose-dominant nectars in the field. However, it is well known that honey bees display a genetic propensity for proboscis response to sucrose [27,28]. 

The colour spectrum experienced by various insects differs from that seen by humans, and the colour perception in many Hymenoptera extends into the ultraviolet range [29]. Therefore, different levels of ultraviolet reflectance may affect the attraction of honey bees and other insects to flowers. Furthermore, carrots have flowers grouped together in large umbels, which provide a large, highly visible landing platform. As umbel size is known to vary between cultivars and individuals, with the primary or terminal umbel being the largest of the umbels on each plant, typically between 100 and 150 mm in diameter [30], both umbel size and UV absorbance may be important for pollinator attraction. 

To date, the few studies that have been conducted on the floral traits that attract insects to different carrot flowers have mainly focussed on floral volatiles from a limited number of cultivars [7,8]. In this study, insect visitation to 17 cultivars of flowering CMS carrots is recorded to determine whether some cultivars attract more insect visitors than others and whether there is variation in the visitation frequency by some of the more abundant visitor groups. The aims are to identify (1) how rates of insect visitation vary to different CMS carrot genotypes or cultivars, and (2) how visitation by honey bees and other insects is related to key floral traits other than floral volatiles, including ultraviolet reflectance, nectar production, and the size of inflorescences. 

## 2. Materials and Methods

### 2.1. Insect Visitation to Seventeen CMS Cultivars

In season 1, a comparison of insect visitation to 17 cultivars (lines) of CMS carrots was conducted in field trial A at Bejo Seeds in Southern Tasmania (42.704° S, 147.445° E), on four days between 14 and 24 December 2002 (24 umbels per cultivar, 408 total umbels observed). The carrot cultivars were planted for a commercial grow-out trial in a completely randomised design (Figure S1). Seventeen carrot lines were allocated to these plots. Of the additional seven plots, six contained duplicated carrot lines not used in this study, and one was empty. Each plot was a 5 m × 1.6 m bed containing three rows of carrots. The rows of carrots in each bed were 0.3 m distant from each other with a 60 cm space between each of the different carrot lines. No managed European honey bee hives were placed at the trial site to prevent positional biasing of the results.

The 17 carrot cultivars were of four different CMS types (Table 1). The two main groups were: brown anther (having non-productive anthers, which are brown in colour) and petaloid (having petal-like structures instead of anthers) with petaloid colour (green, white, or purple) used to subdivide this group into a further three CMS types. There were five brown anther cultivars and three petaloid cultivars: purple (1 cultivar), white (7 cultivars), and green (9 cultivars). All root types were represented by one or more cultivar: Nantes (5 cultivars, n = 120 umbels), Berlicumer (3 cultivars, n = 72 umbels), Berlicumer/Flakee (2 cultivars, n = 48 umbels), Chantenay (2 cultivars, n = 48 umbels), Imperator (2 cultivars, n = 48 umbels), Amsterdam (1 cultivar, n = 24 umbels), Flakee (1 cultivar, n = 24 umbels), and ABK/N (1 cultivar, n = 24 umbels). Details of the cultivars used, and the experimental ID codes allocated to each can be found in Table 1.

Receptive carrot umbels from the 17 cultivars were randomly selected with all selected umbels having > 40% receptive flowers. Each of the four days was divided into three 2-hour observation periods; being 09:00–11:00, 11:00–13:00 and 13:00–15:00. All four observation days were calm and sunny, air temperatures were above 15 °C at 09:00, and the maximum temperature varied between 20 and 27 °C. From each cultivar, two observers each watched two umbels, which were randomly selected and observed for five minutes within each of these time periods. Cultivars were observed in a pre-determined random order. Prior to the commencement of each 5-minute observation period, the number and type of insects that were already present on each of the selected umbels were recorded. 

Each time an insect alighted on one of the umbels under observation, it was recorded according to its classification. No distinction was made between insects alighting for the first time on an umbel or returning to an umbel after departing. Each umbel of a pair was scored separately. To avoid affecting the behaviour of the insects under observation, and to ensure not to cast a shadow over the umbel, the observer remained at least 1 m away from the umbels under observation. The insect groupings recorded were European honey bee (*A. mellifera*)*,* nectar scarabs (predominantly *Phyllotocus rufipennis* (Boisduval)), bee flies (Bombyliidae, predominantly *Comptosia ocellata* (Newman)), flies (Muscoidea predominantly Calliphoridae), native bees (Halictidae and Colletidae), soldier beetles (Cantharidae, specifically *Chauliognathus lugubris* (Fabricius)), wasps, and other, which comprised species that did not fall within the aforementioned groupings.

In field trial A, visitation data for cultivars were also grouped for analysis based on CMS type (brown anther or petaloid), root type, and flower colour to assess the effects of floral morphological differences on insect visitation. Comparisons were undertaken using non-parametric Kruskal–Wallis tests, Mann–Whitney U tests, and Friedman tests, depending on the numbers of cultivars being compared for all insects pooled together and for each insect grouping using SPSS^®^ 17.0. Where Friedman’s and Kruskal–Wallis tests were undertaken, multiple Wilcoxon signed ranks tests were then used to determine which cultivars differed from one another. P values were adjusted using a sequential Bonferroni procedure to control for an increased chance of type-I-error resulting from multiple comparisons.

### 2.2. Floral traits and insect visitation

Ultraviolet reflectance, nectar sugar levels, and umbel diameter are all possible floral traits that may influence insect visitation to an umbel. To examine whether these traits varied among cultivars, field trial B was undertaken. In this trial, umbel insect visitation was recorded in the field, and then, the floral traits of those umbels were assessed. This was performed by selecting six petaloid cultivars that were both commercially available and deemed either the most attractive (PA1, PB3, PF1) or least attractive (PN3, PC2, PBF1) to insect visitors during field trial A. 

Field site B was planted in a randomised complete block design comprising five blocks containing each of the six cultivars (Figure S2). One experimental plot of cultivar PA1 failed to germinate, leaving only four blocks containing PA1 at flowering. Each block consisted of a single bed measuring 10 m × 0.8 m containing three rows of each cultivar. A single bed (3 rows) of one MF cultivar (MX1) was planted in between each of the experimental CMS cultivars to separate the plots and to stimulate pollinating insects, as is the practice in commercial crops. Field trial B was conducted from 20 January to 16 February 2004, at the University Farm (42.797° S, 147.426° E), Southern Tasmania. The methods for the collection and analysis of data for each floral trait are presented in the following sections. 

### 2.2.1. Insect Activity and Diameter

As in trial A, insect visitation was recorded in trial B, but in this trial, the diameter was also measured for each umbel observed. Observations were done each day across all cultivars on seven separate days (n = 160–214 observations per cultivar). Insect activity on each umbel was observed for five minutes. Cultivars were observed in a pre-determined random order. Each time an insect alighted on one of the umbels under observation, it was recorded according to its classification as categorised in trial A. Following observations for insect visits, the diameter of each umbel was measured in the field, by holding the hand flat, underneath the umbel, with fingers on either side of the stem to fully extend the surface area. Then, a 30-cm ruler was used to measure the umbels to the nearest 0.5 cm. Overall, 1198 umbels were observed for insect visits and measured for umbel diameter. Both correlation and direct logistic regression were used to assess the impact that cultivar and umbel diameter had on insect visitation by each grouping, with visitation recorded as visited or not visited.

### 2.2.2. Ultraviolet Reflectance

Photographs of ultraviolet reflectance were taken at field site B. Seven umbels were subsampled per cultivar, removed, and placed in a box. The box sheltered the sample from any wind that may have caused movement of the umbels during photography. Then, the stalk of each umbel was placed directly into a small hole in a black card for photographing. Photographs were taken using a Pentax SP1000 SLR camera (F 5.6) (Tokyo, Japan) and 35 mm Fuji RTP II film (Fujifilm, Tokyo, Japan). This film has enhanced sensitivity to ultraviolet light. A Hoya U360 (ultraviolet bandpass) (Hoya, Tokyo, Japan) filter was used. Illumination was provided by natural sunlight and a Yuzo DC2814 ring flash (Fujifilm, Tokyo, Japan). Three flashes were found to provide optimum exposure to detect ultraviolet reflectance. The camera was mounted on a tripod and focussed prior to attaching the ultraviolet filter. The box and camera were placed at a fixed distance from each other so that refocussing was not required. 

Follow-up confirmation of field photographic results was performed in the laboratory on the cut umbels using a Zeiss Tessovar macro lens system (Carl Zeiss, Oberkochen, Germany) linked to a Watec WAT202D digital colour camera (Watec, Yamagata, Japan). Images were viewed using Fly Video software on a PC fitted with a Fly Video ’98 video capture card (Animation Technologies, Hsin Tien City, Taiwan). 

### 2.2.3. Soluble Sugar Analysis of Nectar

Soluble sugar analysis was undertaken in trial B immediately following photography for UV reflectance. An extraction technique was devised that could be used under windy field conditions by modifying a method by Manetas and Petropoulou [31]:

A carrot umbel consists of four whorls of umbellets. It proved more practical to sample nectar from umbellets rather than entire umbels, with preliminary experiments revealing that dipping carrot umbellets in distilled water 20 times removed over 88% of the nectar. Forceps were used to remove two umbellets from opposite sides of the 3rd whorl of each umbel.

The umbellets were gently dipped 20 times each in bottles containing 10 mL of refrigerated, distilled water. The bottles were sealed and stored at 4 °C for subsequent laboratory analysis. For soluble sugar analysis, seven flowers per cultivar were sampled. Soluble fructose, glucose, and sucrose were identified using high performance liquid chromatography–mass spectrometry (HPLC–MS). The column was a Waters High Performance Carbohydrate Cartridge (Waters, Milford, ME, USA), 4.6 mm × 250 mm, fitted with a guard cartridge of the same material. The mobile phase was 75% methanol/25% water, isocratic at 1.2 mL per minute. Sugars were detected in negative mode by Atmospheric Pressure Chemical Ionisation (APCI) mass spectrometry on a Finnigan LCQ ion trap MS (Thermo Fisher Scientific, Waltham, MA, USA). 

Sugars were detected as negative ion adducts formed by post column infusion of 20 µL/min of 5% formic acid in water. For the monosaccharides tandem MS was used, with the [M+ formate] anion at *m*/*z* = 225.3 being isolated, fragmented using 25% collision energy, and the subsequent daughter ion at *m*/*z* = 179 being further isolated and fragmented at 25% collision energy with the final products at *m*/*z* = 89, 119, 131, and 143 being used for quantitation. Sucrose quantitation was achieved by selected ion monitoring of the [M + formate] anion at *m*/*z* = 387.3.

Variation in total sugar concentration as well as the proportions of fructose, glucose, and sucrose between cultivars, were assessed using non-parametric Kruskal–Wallis tests with SPSS^®^ 17.0.

## 3. Results

### 3.1. Insect Visitation to Seventeen CMS Cultivars

**Figure 1:**
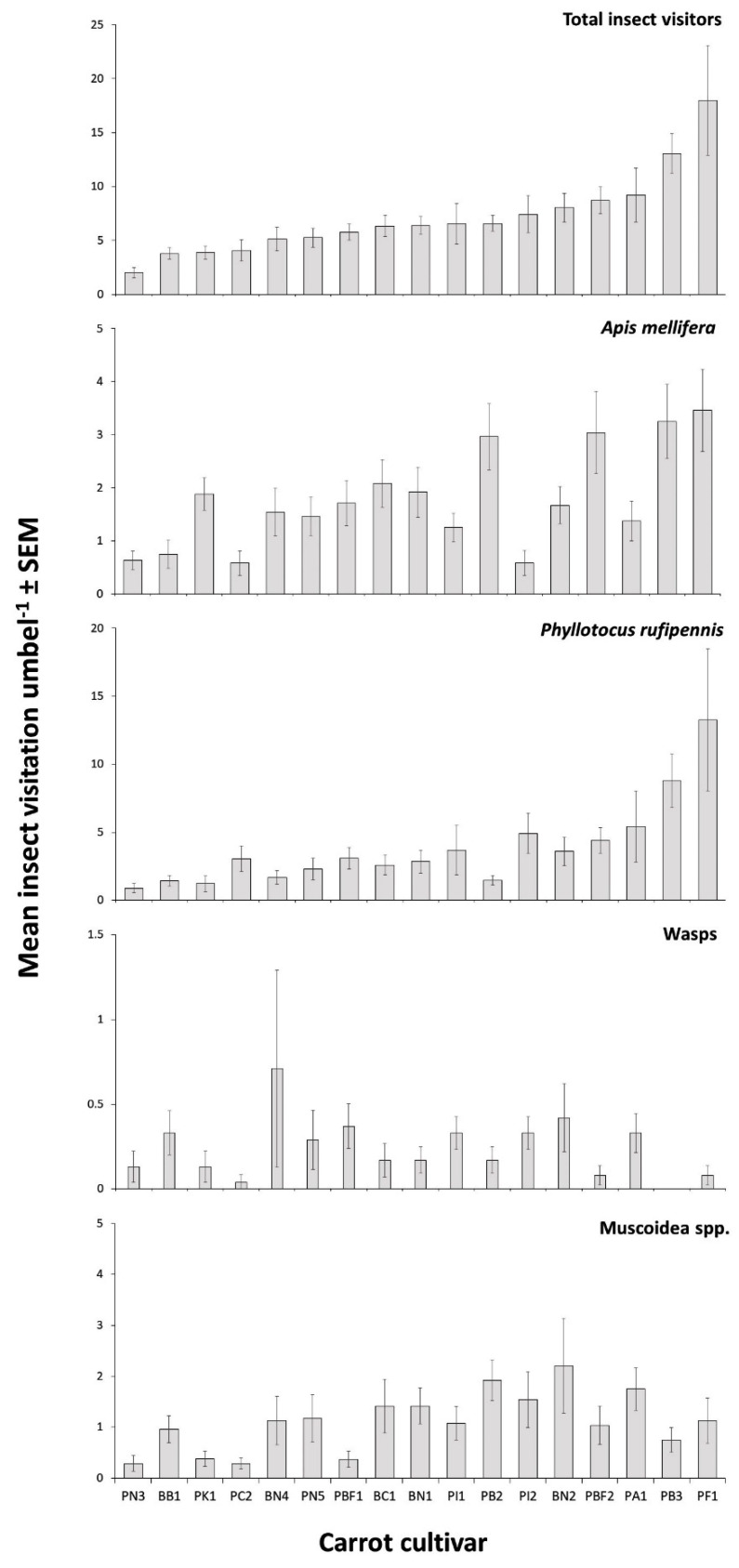
Mean (± SEM) total insect visitors, *Apis mellifera, Phyllotocus rufipennis* (nectar scarabs), wasp, and muscoid fly. Visitation per 5 min to 17 hybrid CMS carrot cultivars in field trial A between January and February (n = 24 umbels per cultivar).

Observations of insects in field trial A revealed that some carrot cultivars attracted more insects than others (Figure 1). Total insect visitation was found to be significantly different among cultivars (Kruskal–Wallis test χ^2^ = 93.84, df = 1, *p* < 0.001, Figure 1). There were 2877 insect visits, of which 97.8% were from four groups; 53.8% were from nectar scarabs, 25.1% were from honey bees, 15.5% were from muscoid flies, and 3.4% were from wasps. 

Total insect visitation was found to be significantly different among root types (Kruskal–Wallis test χ^2^ = 42.31, df = 7, *p* < 0.001) with Berlicumer root cultivars being visited by significantly more insects (7.8 ± 0.8 visits per 5 min) than Nantes (5.4 ± 0.5 visits per 5 min) (Mann–Whitney test U = 3109, *p* = 0.0011).

Visitation rates among cultivars with flower colours of green, white, and purple were not found to be significantly different (Kruskal–Wallis test χ^2^ = 5.03, df = 2, *p* = 0.081). Similarly, visitation rates among cultivars of different CMS type (brown anther and petaloid) were not found to be significantly different (Mann–Whitney test U = 16554, *p* = 0.502, brown anther n = 120, petaloid n = 288) with a mean of 5.45 (SEM = 0.35, median = 3) insects visits per 5 min for the brown anther cultivars compared to a mean visitation rate of 4.21 (SEM = 0.47, median = 3) insect visits per 5 min for the petaloid cultivars.

Visitation was found to be significantly different within the four main insect groups (Figure 1). Honey bees (Kruskal–Wallis test χ^2^ = 41.44, df = 16, *p* = 0.001), nectar scarabs (*P. rufipennis*) (Kruskal–Wallis test χ^2^ = 44.91, df = 16, *p* = 0.001), muscoid flies (Kruskal–Wallis test χ^2^ = 34.01, df = 16, *p* = 0.0054), and wasps (Kruskal–Wallis test χ^2^ = 27.80, df = 16, *p* = 0.033) were all found to have significantly different visitation rates among the 17 different carrot cultivars. Both honey bees and nectar scarabs showed some concordance in their broad rankings of cultivar visitation frequencies. 

### 3.2. Umbel Size, Cultivar, and Insect Visitation

**Figure 2:**
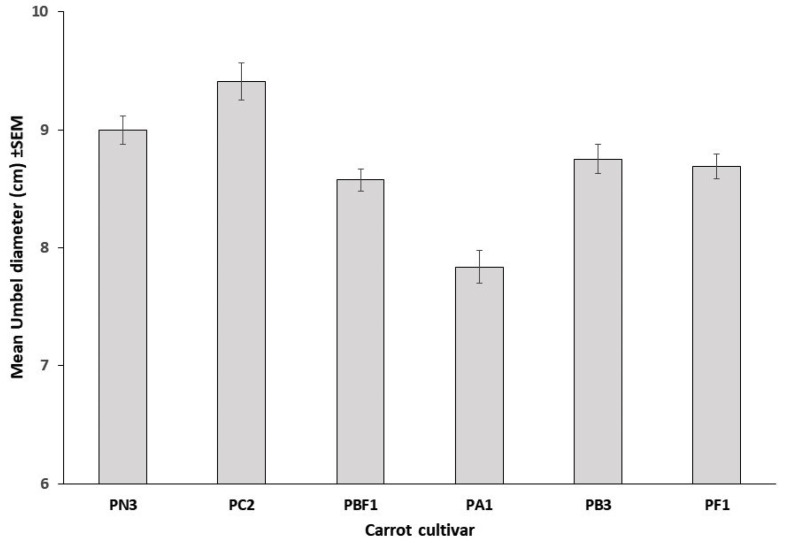
Mean (± SEM) umbel diameters of six hybrid CMS carrot cultivars assessed in field trial B (n = 210, 208, 160, 202, 204, 214).

Umbel diameter differed significantly among cultivars (F_5,1192_ = 15.82, *p* < 0.0001; Figure 2). Furthermore, when grouped by the umbel diameter, the unattractive cultivars (mean ± SEM: 9.11 ± 0.36 cm) were significantly larger (t_34_ = −3.436, *p* = 0.002) than the attractive cultivars (7.56 ± 0.28 cm). The composition of insect visitors in trial B differed to that in trial A. There were 1610 insect visits in trial B with 25.7% of visits being soldier beetles, as well as 20.2% honey bees, 12.6% bee flies, 11.9% muscoid flies, 10.8% ladybirds, 4.6% wasps, 3.1% native bees, and only 2.4% nectar scarabs. For soldier beetles, the inclusion of cultivar and diameter into predictive models using direct binary logistic regression, indicated both diameter (Wald = 19.14, df = 1, *p* < 0.001) and cultivar (Wald = 15.82, df = 5, *p* = 0.007) to be significant predictors of soldier beetle visitation, with a 1.2-fold increase in the likelihood of visitation with every centimeter increase in umbel diameter. For honey bees, both diameter (Wald = 17.74, df = 1, *p* < 0.001) and cultivar (Wald = 30.86, df = 5, *p* < 0.001) were significant with a 0.81-fold decrease in the likelihood of honey bee visitation with every centimeter increase in umbel diameter. However, by taking into account the number of soldier beetles on flowers prior to observing insect visitation, a significant effect of soldier beetle presence on honey bee visitation was evident, with an increase in model fit by 15% and a 0.47-fold decrease in the likelihood of honey bee visitation (Wald = 4.92, df = 1, *p* = 0.026) with every soldier beetle initially present on flowers. Soldier beetles initially present on flowers ranged from zero to three beetles per flower with 7.7% (n = 1198) of all flowers scored having soldier beetles present on them at the time of the first observation. Neither ladybird (Wald = 9.85, df = 5, *p* = 0.08) nor native bee (Wald = 9.71, df = 5, *p* = 0.08) visitation showed a significant relationship with cultivar. However, both ladybird (Wald = 6.96, df = 1, *p* = 0.008) and native bee (Wald = 6.62, df = 1, *p* = 0.01) visitation increased marginally but significantly with increased umbel diameter. The logistic models described correctly predicted insect visits 78.5–96.7% of the time.

### 3.3. Floral Ultraviolet Reflectance and Nectar Sugars

**Figure 3:**
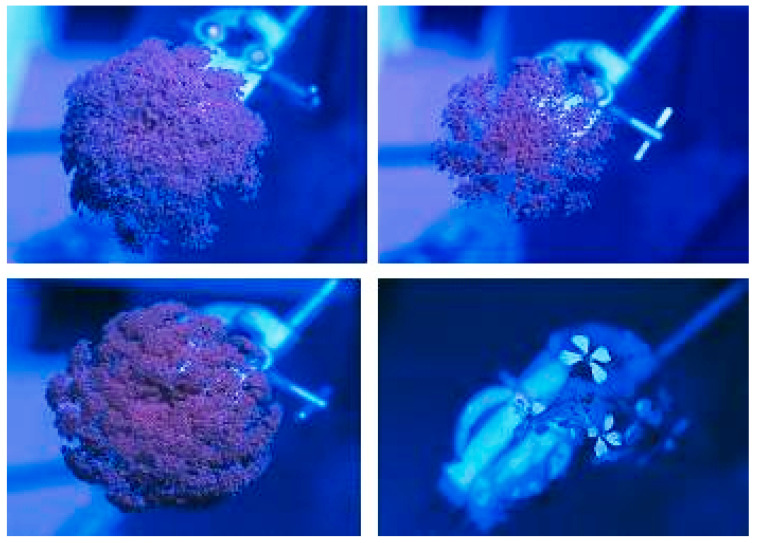
Comparison of ultraviolet reflectance of *Daucus carota* (3 replicates) vs. *Raphanus raphanistrum* (wild radish)—bottom right—when photographed under field conditions. Ultraviolet reflectance appears white.

No ultraviolet reflectance was seen on any of the carrot umbels photographed in the field (Figure 3). Test photographs revealed ultraviolet reflectance in other plants, such as wild radish, but not in carrot flowers.

**Figure 4:**
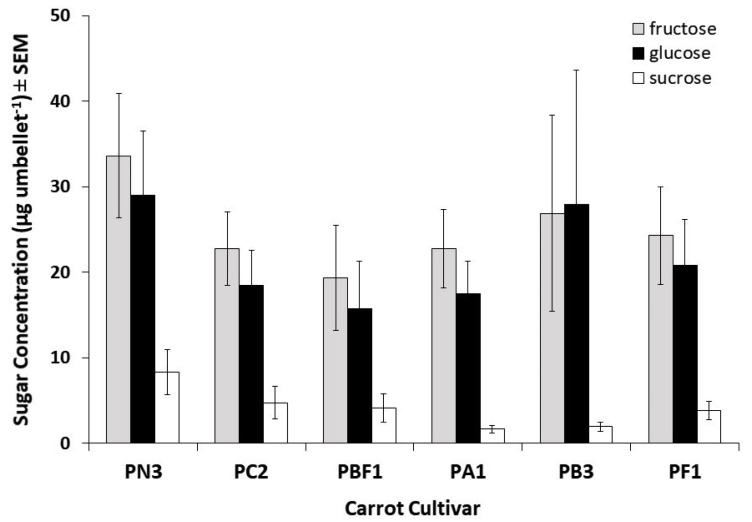
Fructose, glucose, and sucrose content (mean ± SEM) of nectar collected from six hybrid CMS carrot cultivars observed for insect visitation during trial B (n = 7 per cultivar).

The nectar from the carrot cultivars used in this trial was found to be dominant in monosaccharides (fructose and glucose, Figure 4). Sucrose, a disaccharide, was also present in nectar samples from all cultivars varying between 7% and 29% of the monosaccharide levels and accounted for approximately 8% of the overall total sugars collected. The proportions of the individual sugars varied greatly among umbellets within cultivars; for example, in cultivar PB3, fructose concentrations varied between 80.7 and 945.1 µg per umbellet, glucose varied between 54.3 and 1219.8 µg per umbellet, and sucrose varied between 1.8 and 42.2 µg per umbellet. Although variation was observed in total sugar concentrations as well as in the proportions of fructose, glucose, and sucrose between cultivars, there was no significant difference between cultivars in fructose (Kruskal–Wallis test χ^2^ = 3.28, df = 5, *p* = 0.66), glucose (Kruskal–Wallis test χ^2^ = 2.86, df = 5, *p* = 0.72), sucrose (Kruskal–Wallis test χ^2^ = 5.25, df = 5, *p* = 0.39), or total sugar content (Kruskal–Wallis test χ^2^ = 3.01, df = 5, *p* = 0.70; Figure 4). Similarly, when grouped by attractive and unattractive cultivar, no significant difference was found in sucrose (U = 133, *p* = 0.359), fructose (U = 156, *p* = 0.849), glucose (U =154, *p* = 0.800), or total sugar concentration (U = 162, *p* = 1.00).

## 4. Discussion

Insect-pollinated crops require reliable and effective insect visitation to maximise seed set and yield. In this study, significant differences in insect visitation rates were found among CMS carrot cultivars in field trials. In the initial trial involving 17 cultivars across eight root types and four CMS systems, a greater than threefold difference in overall visitation rate between the least and the most visited cultivars was found, confirming the results from previous trials that some cultivars of CMS carrots appear to be more attractive to pollinators, including honey bees, than others [13,14,15,31]. Visits of honey bees in our first trial varied among cultivars from 0.58 ± 0.23 to 3.46 ± 0.23 visits/umbel/5 min.

In the current study, more insects visited cultivars with the Berlicumer than the Nantes root types. While there are no reported comparable studies on the effect of carrot root type on pollinator attraction, we suggest that the observed difference between Berlicumer and Nantes demonstrates a genotypic influence on pollinator visitation. We found no significant difference in insect visitation between brown anther and petaloid phenotypes. In a previous study of three brown anther cultivars, one white petaloid cultivar, and one light green petaloid cultivar, brown anther cultivars were reported to be more attractive to honey bees than white petaloid types [15]. Furthermore, Erickson *et al*. found that honey bees show intra-phenotypic and genotypic foraging preferences between white and light green flowers in open-pollinated carrot cultivars [14]. In our study, the lack of difference in visitation rate between CMS types indicates that differences in physical appearance associated with male sterility traits do not influence pollinator behaviour in this case. It is possible that root type and flower colour do not directly impact pollinator visitation, but instead, these traits could occasionally be genetically linked to other physiological traits, such as the production of nectar and volatiles, which would affect pollinator foraging behaviour and would explain the variation in results between studies.

Significant differences in floral diameters were evident among cultivars. However, when grouped into attractive and unattractive cultivars, although a significant difference in umbel diameter was observed, the unattractive cultivars were shown to produce the larger umbels. The influence of umbel diameter in this study varied according to the insect species, with more soldier beetles visiting larger umbels, and more ladybirds and native bees visiting smaller umbels. Other studies have also found that flower size affects the behaviour of pollinators. Abraham found that naïve bumblebees (*Bombus* spp.) visited all of the flowers of 15 Althea (*Hibiscus syriacus* L.) plants in equal numbers but visited the larger flowers first [32]. It was suggested that this was an optimal foraging strategy, as larger flowers were likely to produce more nectar. Similarly, on *Achillea ptarmica* (Asteraceae), it has been shown that the pollinating insects visited larger, many-headed inflorescences in preference to inflorescences with less floral heads [33]. 

We found that honey bee visits decreased with increasing umbel diameter and/or if soldier beetles were initially present on the umbel. Such interference from other insects was also found by Danderson and Molano-Flores, who found that larger floral displays on *Eryngium yuccifolium* (Apiaceae) attracted more visitors, but that herbivory on inflorescences negatively affected pollinator visitation [34]. Furthermore, Kirk *et al.* found that nectar-foraging honey bees were deterred from landing on umbels with real or simulated pollen beetles *Meligethes aeneus* (Nitidulidae) present [35]. It is plausible, given the negative relationship between the presence of *C. lugubris* and *A. mellifera* and the preference shown for smaller umbels by *A. mellifera,* that it was the presence of *C. lugubris* that deterred *A. mellifera* from preferentially visiting larger umbels. 

Although the structure of carrot inflorescences is in the form of large, highly visible, white or pale green umbels, which would provide a conspicuous visible cue for insects seeking nectar, there is no evidence that colour affects insect visitation. In addition, no significant levels of UV reflectance were observed; only small pinpricks of UV reflectance from nectar were seen in some of the carrot flowers. 

In general, male sterile carrot flowers (not producing pollen) are the least visited [3], warranting the analysis of nectar as the likely most important reward. However, our analysis indicated that nectar composition varies greatly between umbellets within cultivars but not significantly among cultivars. Furthermore, the nectar composition was dominated by monosaccharides, with only small amounts (2–12%) of sucrose. Such a mixture, rich in monosaccharides, can be predicted to be less than ideal for honey bee attraction, with species that are best pollinated by honey bees, generally found to be dominant in sucrose [24,25]. Our findings are not unexpected, as Apiaceae are known to have nectar with relatively low sucrose levels. We found that two of the least attractive cultivars assessed here contained both the highest and lowest total sugar concentrations (mean ± SEM: PN3: 70.9 ± 17.1 µg per umbellet; PBF1: 39.2 ± 12.9 µg per umbellet), indicating that sugar content within the nectars had little effect on pollinator visitation. In earlier studies, it has been found that *Carum carvi* (annual caraway: Apiaceae), had just 13.6% sucrose in its nectar [36], and *Eryngium campestre* (field eryngo), *Scandix australis* (southern shepherd’s needle), *Thapsia garganica,* and *Tordylium apulum* (Mediterranean hartwort) are also low in sucrose with an average of only 8.8% of the total sugar content [37]. In the latter study, it was found that plants with high disaccharide/monosaccharide ratios were attractive to bees and wasps, whereas those with the opposite composition, similar to the carrot cultivars in our study, were more attractive to syrphids (hoverflies), anthomyids (root flies), and beetles.

The results from our investigation indicate that umbel colour and nectar sugar composition appear to be of little importance regarding pollinator attraction to hybrid CMS carrot umbels. Such a result strongly suggests that other factors, most likely of chemical origin, are at play. Floral volatiles have been proposed to be important for pollinator attraction in many systems [38,39], including hybrid carrot [4,7,8]. Chemical components in nectar, apart from fructose, glucose, and sucrose, have also been highlighted as candidates for pollinator attractants [22,40]. For fertile lines, even pollen properties may be important in the decision making of pollinators [41,42]. In hybrid carrot pollination, no direct links among any variable chemical factors from the flower, nectar or pollen, whether volatiles, phenolics, sugars, or minerals, have been reported. We believe more detailed studies on the chemical ecology of hybrid carrot flowers are necessary to reveal which factors are driving the low pollinator attraction observed in hybrid carrot systems. Such an understanding would allow the development of practical breeding strategies to rectify the poor pollinator visitation and seed yields observed. 

## 5. Conclusions

In conclusion, observations of pollinator visitation to hybrid carrot cultivars have shown major differences in attraction among cultivars. The colour of the flowers does not appear to affect attraction, and no ultraviolet reflectance was observed. Umbel size and/or soldier beetle presence affected honeybee visitations. Among all tested cultivars, the nectar was rich in monosaccharides and poor in sucrose; large variations between individual umbels were found, but there were no significant differences among cultivars that could explain the variability in attraction.

## Figures and Tables

**Table 1:** Descriptions of the 17 carrot cultivars and cytoplasmically male-sterile (CMS) plant types used in field trial A.

Cultivar ID	Root Type	CMS Type	Colour	
BN4	Nantes	Brown Anther	White	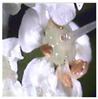 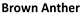 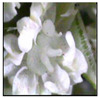 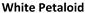 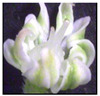 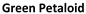 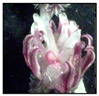 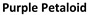
BB1	Berlicumer	Brown Anther	White	
BN1	Nantes	Brown Anther	White	
BN2	Nantes	Brown Anther	White	
BC1	Chantenay	Brown Anther	White	
PC2	Chantenay	Petaloid	White	
PF1	Flakee	Petaloid	White	
PA1	Amsterdam	Petaloid	Light Green	
PI1	Imperator	Petaloid	Light Green	
PI2	Imperator	Petaloid	Light Green	
PN3	Nantes	Petaloid	Light Green	
PN5	Nantes	Petaloid	Light Green	
PB3	Berlicumer	Petaloid	Light Green	
PB2	Berlicumer	Petaloid	Light Green	
PBF1	Berl/Flakee	Petaloid	Light Green	
PBF2	Berl/Flakee	Petaloid	Light Green	
PK1	ABK/N	Petaloid	Purple	
MX1		Male-fertile	Light Green	

## Supplementary Materials

The following are available online at https://www.mdpi.com/2075-4450/11/7/402/s1, Figure S1: Field trial a experimental layout, commercial grow-out trial., Figure S2: Field trial B experimental layout, commercial grow-out trial.
