# Supplementary material for: It Is Not All about Being Sweet: Differences in Floral Traits and Insect Visitation among Hybrid Carrot Cultivars
Source: Insects. 2020 Jun 29;11(7):402. doi: 10.3390/insects11070402 (PMC7412318; doi:10.3390/insects11070402)
Supplement: Supplementary file 1 [file insects-11-00402-s001.pdf]

It is not all about being sweet: Differences in floral traits and insect visitation among hybrid carrot cultivars

Ann Gaffney, Björn Bohman, Stephen R. Quarrell, Philip H. Brown and Geoff R. Allen

Supplementary information

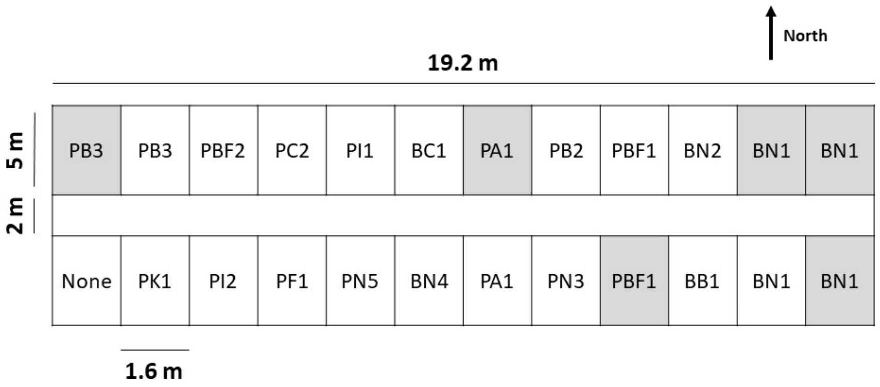

Figure S1. Field trial A experimental layout, commercial grow-out trial. Shaded blocks were not utilized during the experimental period. Whole site area = 230 m^2

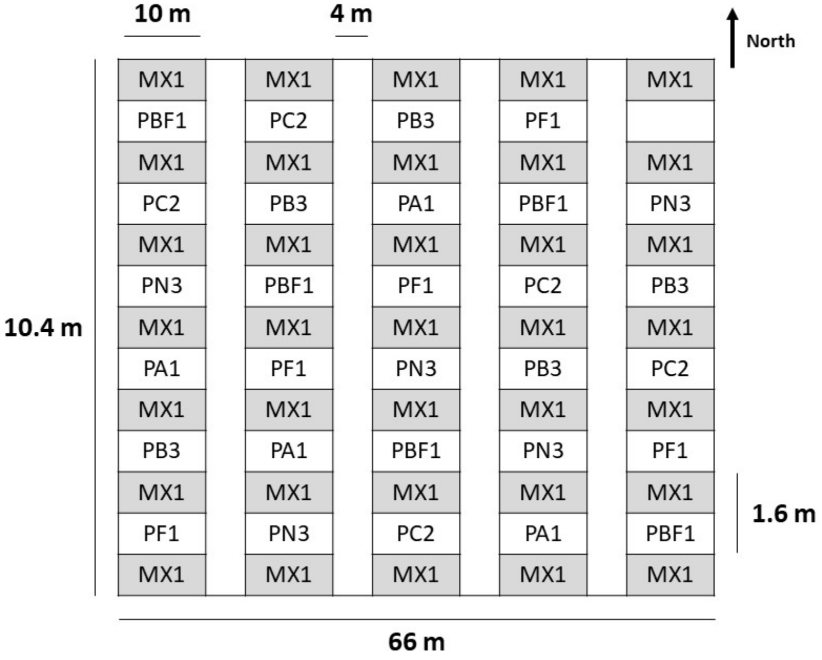

Figure S2. Field trial B experimental layout, commercial grow-out trial. Whole site area = 686 m^2
